# Efficient Electrocatalyst Nanoparticles from Upcycled Class II Capacitors

**DOI:** 10.3390/nano12152697

**Published:** 2022-08-05

**Authors:** Junhua Xu, Daobin Liu, Carmen Lee, Pierre Feydi, Marlene Chapuis, Jing Yu, Emmanuel Billy, Qingyu Yan, Jean-Christophe P. Gabriel

**Affiliations:** 1 SCARCE Laboratory, Energy Research Institute @ NTU (ERI@N), Nanyang Technology University, Singapore 637553, Singapore; 2 Nuclear Chemistry & Separation and Purification Technology Laboratory, Fujian Institute of Research on the Structure of Matter, Chinese Academy of Sciences, Fuzhou 350002, China; 3 LITEN, Université Grenoble Alpes, CEA, 38054 Grenoble, France; 4 School of Materials Science and Engineering, Nanyang Technological University, Singapore 639798, Singapore; 5 LICSEN, NIMBE, Université Paris-Saclay, CEA, CNRS, 91191 Gif-sur-Yvette, France

**Keywords:** electrocatalysis, nickel, electronic waste, layered double hydroxide, recycling, circular economy, re-use, nanoparticle, liquid-liquid extraction, ionic liquid, ceramic capacitor

## Abstract

To move away from fossil fuels, the electrochemical reaction plays a critical role in renewable energy sources and devices. The anodic oxygen evolution reaction (OER) is always coupled with these reactions in devices but suffers from large energy barriers. Thus, it is important for developing efficient OER catalysts with low overpotential. On the other hand, there are large amounts of metals in electronic waste (E-waste), especially various transition metals that are promising alternatives for catalyzing OER. Hence, this work, which focuses on upcycling Class II BaTiO_3_ Multilayer Ceramic Capacitors, of which two trillion were produced in 2011 alone. We achieved this by first using a green solvent extraction method that combined the ionic liquid Aliquat^®^ 336 and hydrochloride acid to recover a mixed solution of Ni, Fe and Cu cations, and then using such a solution to synthesize high potential catalysts NiFe hydroxide and NiCu hydroxide for OER. NiFe-hydroxide has been demonstrated to have faster OER kinetics than the NiCu-hydroxide and commercial c-RuO_2_. In addition, it showed promising results after the chronopotentiometry tests that outperform c-RuO_2_.

## 1. Introduction

We are currently facing a major environmental crisis, creating the need to drastically reduce our emission of greenhouse gases, which requires a move away from fossil fuels towards renewable energy sources and devices, such as hydrogen production and CO_2_ reduction via the electrochemical method. Noteworthily, an electrochemical oxygen evolution reaction (OER) is a typical reaction to couple with these cathodic reactions, which requires a large overpotential to conquer the energy barriers. Thus, it is important for developing efficient OER catalysts [1,2]. This should be done in a sustainable manner. Hence, what about using our own wastes toward that goal? Indeed, todays connected consumer’s economic model creates a huge amount of electronic waste (E-waste), approximately 50 million tons per year worldwide. This is an alarming issue because this stream of waste increases rapidly every year. Most importantly, such E-wastes consist of a large variety of materials and components that include toxic and critical ones, which represent a major challenge in terms of waste management. Recovered chemical elements of E-waste have an estimated potential value of approximately 50 to 60 billion € depending on stock exchange materials prices, but currently, only 20% of E-waste is recycled in certified processes [3].

Looking into the area of recycling of Printed Circuit Boards (PCBs), it is understood that their recycling has had limited success. Hence, of the sixty chemical elements classically found in a PCB, only a fraction of them was successfully recycled [4,5]. Pyrometallurgy is the most common method for their recycling, as it can recover approximately ten chemical elements with success, which accounts for ~30 w% of the total PCB’s mass [6]. The remaining components are either burned into fumes (for the organic phase) or mixed in with the fly and bottom ashes. The disadvantages of this process lie in the needs for large scale smelters and for a centralized PCB waste process plant to collect them from all over a wide geographic zone. Therefore, there is a demand for alternative processes that: (i) enable better elemental separation, especially for elements with higher atomic numbers, better recovery rates and valorization; (ii) are more environmentally friendly; (iii) can be deployed in small countries with small volumes of waste; (iv) do not burn epoxy resins, which releases toxic fumes and greenhouse gases [7].

The primary focus of our research group is on the recycling of Printed Circuit Boards (PCBs), with the strategy of dismantling PCBs first [8,9], followed by an efficient protocol to sort the individual components into different categories, for example, bare boards, solder, CPUs and capacitors [10,11]. Each category will comprise components with much simpler overall elemental compositions, which will make recycling treatment relatively easy for reuse purposes, and will reduce the chemical variability of process inputs [12]. One such category would contain Multilayer Ceramic Capacitor (MLCC) components that are commonly seen in PCBs. Indeed, it is estimated that two trillion MLCCs were produced in 2011 [13,14]. Many of them are Class II capacitors, as they are made of alternating layers of high dielectric constant (K) materials (typically between 1000 to >20,000) and metal (nickel or copper or tin) layers as electrodes. Such dielectric materials are typically made of the ferroelectric ceramic BaTiO_3_ [13].

Solvent extraction is a classic separation technique, which has been widely used for separation and purification of metals in the laboratory and industry [15]. It classically involves the use of organic solvents, which lead to environmental problems, as they have the disadvantages of being volatile, flammable and often toxic [16]. Hence, the necessity to employ environmentally friendly solvents, such as ionic liquids, to replace traditional organic extractants for metal extraction and separation. Ionic liquid solvents consist entirely of molecular ions that have the advantages of a broad liquid range, a negligible vapor pressure and low flammability [17]. Likewise, quaternary ammonium extractant Aliquat 336 is liquid at room temperature and can be utilized in an undiluted form as the organic extractant, hence avoiding the use of volatile organic solvents as diluents. It has previously been shown as a very efficient extractant for the separation and purification of transition metals and rare earths [18,19,20].

In this paper, we first report on the combination of leaching and extraction processes into a single step, and the subsequent recovery of Ni, Fe and Cu elements from Class II capacitors (BaTiO_3_ MLCCs), using Aliquat 336 and hydrochloric acid, thereby leading to concentrated Ni^2+^, Fe^2+^ and Cu^2+^ ions solutions. Then, we demonstrate the upcycling potential of such recovered metallic solutions with their use for the synthesis of a highly efficient amorphous anode catalyst for the electrolysis of water.

## 2. Materials and Methods

Hydrochloride acid (HCl, 37%), Aliquat^®^ 336 (88.2–93.0% quaternary content) (Thermo Scientific, Singapore), ammonia (25%), nickel chloride (NiCl_2_, 99%), iron chloride (FeCl_3_, 99%), sodium hydroxide (NaOH, 97%), sulfuric acid (98%), nitric acid (HNO_3_, 70%) and propylene oxide (PO) were used. All chemicals were purchased from Sigma-Aldrich (Waltham, MA, USA) and used as received without further purification. Milli Q water (18.2 MΩ·cm) from a water purification system (WaterPro^®^, Labconco Co., Kent Town, Australia) was employed in all syntheses. 

The XRD patterns were obtained using the Bruker D8 Advanced X-ray diffractometer equipped with a Cu–Kα radiation source (λ = 1.5406 Å). The analysis was conducted at 40 kV and 40 mA in a 2-θ range between 10° and 80°. Scanning electron microscopy (SEM–EDS, JSMIT300, Japan Electronics Corporation) was used to analyze morphology and element detection. The samples were coated with a layer of carbon via sputter deposition (SPI-MODULETM Carbon Coater and Control) before being loaded into the SEM, and the electron gun voltage was set to 20 kV. ICP-OES was used to determine the compositions that were present in the extracted aqueous samples. To analyze the elemental composition of the samples, an ICP-OES spectrometer PerkinElmer Optima 8000 was used (Waltham, MA, USA); all data from ICP-OES employed in this article were within 5% error. X-ray photoelectron spectroscopy (XPS) measurements were conducted on a Thermo Fisher ESCALB 250xi (delay line detector) spectrometer equipped with a monochromatic Al Kα X-ray source (1486.6 eV). The C 1s peak at 284.8 eV was used to calibrate the binding energy of all spectra.

In order to evaluate the metals extracted from the liquid zone, the distribution ratio was employed for different liquid zones [21]. Distribution ratios of Ni and Cu between different zones in the multi extraction system were calculated using the following equations.

(1) $$D_{M,\frac{t}{m}}=\frac{C_{M,t}}{C_{M,m}}$$

(2) $$D_{M,\frac{t}{b}}=\frac{C_{M,t}}{C_{M,b}}$$

(3) $$D_{M,\frac{m}{b}}=\frac{C_{M,m}}{C_{M,b}}$$

Here, M refers to the ion in the specific extraction zone, $C_{M,t}$, $C_{M,m}$ and $C_{M,b}$ refer to the concentrations of metal ion M in the top zone, middle zone and bottom zone. 

Moreover, the separation factor (*α*) between Ni and Cu ions was calculated by the ratio of their distribution ratios using the equation below.

(4) $$\alpha_{\frac{Ni}{Cu},t/m}=\frac{D_{Ni\frac{t}{m}}}{D_{\mathrm{Cu},\frac{t}{m}}}$$

**Leaching of MLCCs:** Dissolution of MLCCs for the analysis of metal composition was performed using microwave digestion equipment. Typically, 1.0 g of whole MLCC components was immersed in 10 mL of hydrochloric acid (HCl, 6 M), which was placed in a PTFE acid digestion vessel and mixed before sealing. The sealed vessel was then heated via microwave irradiation up to 210 °C and maintained at this temperature for 15 min. Subsequently, the contents in the sealed vessel were centrifuged (12,160× *g*) for 10 min, using a Velocity 14 centrifuge. This gave rise to: (i) a clear solution where the composition of chemical elements was determined using inductively coupled plasma-optical emission spectrometry (ICP-OES); (ii) a residual solid which was left to dry overnight (mass = 0.61 mg) and analyzed using energy-dispersive X-ray spectroscopy.

**Leaching and Solvent Extraction Studies:** Hydrochloric acid (HCl, 6 M) was added to the IL Aliquat^®^ 336 (1:1 *v*/*v*) in a 15 mL centrifugation tube. Two phases were clearly observed in this step. The upper layer was the IL Aliquat^®^ 336 and the bottom layer was the HCl solution. Next, the entire Class II BaTiO3 Multilayer Ceramic Capacitor (weighing 1.0299 g) was immersed into the mixture and the tube was sealed and agitated at 1000 rotations per minute (rpm) for 5 h at 60 °C, using the Eppendorf ThermoMixer C equipment. Under these conditions, the two phases remained separated and did not form any emulsion. After this step, various domains of different colors were observed. The upper domains comprised the ionic liquid containing dissolved metal ions. These layers were separated out and subsequently back-extracted using three different aqueous solutions: (i) first a HCl aqueous solution (6 M); (ii) then freshly deionized water (pH = 7.0); and finally (iii) an ammonia aqueous solution. For each back-extraction step, one of these aqueous phases was contacted with the extracted ionic liquid solution, in a volume ratio of 1:1. The mixture was then subjected to intensive shaking for a duration of 30 min at 60 °C, then left to decant, and both phases were separated. All recovered back-extraction aqueous phases were then characterized by ICP-OES. Other aqueous solution-based domains were collected separately and diluted for ICP-OES analysis.

**Lead Selective separation:** This experimental procedure aimed to selectively precipitate lead ions from other metallic ions from the HCl (6 M)-based back-extraction solutions. Typically, 5 mL of lead containing aqueous solution were added to a test tube, and a 6 M NaOH solution was subsequently added dropwise to increase the pH up to 12.0, which allowed for some precipitation to occur. After phase separation by centrifugation, the supernatant was transferred to another tube for further analysis, and the solid was washed three times with deionized water. Subsequently, 1 mL of H_2_SO_4_ (1 M) was added to lixiviate the recovered solid precipitate, dissolving most of I; just a small amount of white solid remained. After centrifugation, the supernatant was prepared for further analysis using ICP-OES. 

**Recycling of Used IL:** The recycling of the used IL Aliquat^®^ 336 was performed in two steps, using 6 M NaOH for scrubbing in a volume ratio 1:3, and 0.5 M HCl in a volume ratio 1:2 for regeneration [22]. In the scrubbing step, the IL obtained from the back extraction and 6 M NaOH were mixed together in a tube in equal volumes. The mixture was then agitated at 1000 rpm for 3 h at 60 °C using the Eppendorf ThermoMixer C. Subsequently, the mixture was centrifuged (12,160× *g*) for 10 min. The purpose of this step is to separate the NaOH solution and the ionic liquid. In the regeneration step, the procedures in the scrubbing step were repeated again, using 0.5 M HCl instead of 6 M NaOH. Compared to the initial amount of 5 mL Aliquat^®^ 336, approximately 4.5 mL (90%) of refreshed IL was recovered.

**Synthesis of Multi-metallic Hydroxide Catalyst:** We performed NiFe-hydroxide and NiCu-hydroxide synthesis according to an Ni-LDH reference protocol modified to avoid crystallization of the catalyst [23]. The multi-metallic hydroxide containing Ni and Fe (NiFe-hydroxide) catalyst was synthesized from a simulated leachate (to enable a larger number of studies), which was prepared using a modified Sol-Gel method in aqueous solution. The weight ratios of the chemical precursors, Ni and Fe, were 95.7% and 4.3%, respectively. This ensured that the composition of the simulated leachate was consistent with the composition of the experimental back extraction solution. Typically, 195.9 mg of NiCl_2_∙6H_2_O and 9.8 mg of FeCl_3_ were dissolved into 9 mL of deionized water. Then, 2.6 mL of propylene oxide was added dropwise into the mixture. The mixture was stirred at room temperature for 48 h. Lastly, the solid precipitate formed was collected by centrifugation, followed by thorough washing with ethanol and deionized water; and finally, it was dried in an oven at 60 °C for 2 days. Approximately 57.3 mg of the final product was collected. The yield of NiFe-hydroxide was calculated from the losses of Ni and Fe in the final solution when compared to the initial one (concentrations were measured by ICPOES). Hence, the overall calculated NiFe-hydroxide yield was 80.8%, consuming 80.2% of the initial Ni and 95.5% of the initial Fe. The remaining unreacted Ni and Fe can be directly reused in a new cycle of catalyst synthesis to further increase sustainability. The final NiFe-hydroxide product was then characterized using XRD and SEM-EDX analyses, and its catalytic properties were determined using electrochemical measurements. The NiCu-hydroxide was synthesized using the same procedure as NiFe-hydroxide except the precursor 722.8 mg of NiCl_2_·6H_2_O and 62.42 mg of CuCl_2_. In addition, Ni-LDH was synthesized and characterized similarly to the NiFe-hydroxide synthesis protocol, but without addition of the FeCl_3_ precursor.

**Electrocatalysis measurements:** In the electrochemical cell set-up, a modified glassy carbon disk with a diameter of 5.0 mm (Gaoss Union, Tianjin, China) was used as the working electrode. The following is a description of the electrode’s modification protocol. Firstly, 8 mg of as-obtained catalyst was dispersed in 1 mL of a water/ethanol mixture (1:1 volume ratio). Secondly, 50 µL of 5 wt% Nafion solution (Sigma-Aldrich) was added. The mixture was then sonicated for 2 h to form a homogeneous catalyst ink. Thirdly, the catalyst ink was dropcasted onto the working electrode’s surface. The total catalyst loading on the working electrode was 0.133 mg/cm^2^. Finally, the working electrode with catalyst loading was dried under ambient conditions prior to performing oxygen evolution reaction (OER) measurements.

All electrochemical measurements were conducted at room temperature in a conventional three-electrode electrochemical cell using a 1.0 M KOH aqueous solution as electrolyte and controlled by the Solartron Potentiostat workstation. A saturated Ag/AgCl electrode and a graphite rod were used as the reference electrode and the counter electrode, respectively. All the electrochemical measurement potentials were recalibrated to the potentials with reference to the reversible hydrogen electrode (RHE), using the equation of E(RHE) = E(Ag/AgCl) + 0.059 pH + 0.198. Polarization curves were obtained between 1.0 and 1.7 V (vs. RHE) with a scan rate of 5 mV/s. The electrochemical impedance spectroscopy (EIS) measurements were performed at an applied potential of 1.50 V (vs. RHE) with 5 mV a.c. amplitude from 0.1 to 10^5^ Hz. The stability of NiFe-hydroxide catalyst was evaluated by performing chronopotentiometry for 20 h. 

## 3. Metals Lixiviation and Extraction

First, we enacted a baseline overall metal composition analysis of the Class II BaTiO_3_ MLCC used in this study using a combination of ICP-OES and SEM-EDX: ICP-OES analysis was performed on the leaching solution obtained from the microwave acid digestion of MLCCs recovered from dismantled waste PCBs, and SEM-EDX analysis was performed on the undissolved solid residue. The ICP-OES analysis of the BaTiO_3_ MLCC lixiviate indicated that Ni, Ba, Cu, Sn and Pb were the main metal components, with concentrations of 66.1, 17.4, 8.0, 5.7 and 1.03 wt%, respectively, with other metals making up the remaining percentage (Ti, Al, Mn and Fe) for a total of 1.23 wt%. Additional SEM-EDX analysis was performed on the undissolved solid residue recovered after the microwave digestion (Figure S1, Supplementary Materials), and showed that its metal content consisted mostly of titanium. These results are consistent with previous reports [24,25]. It should be noted that palladium (Pd) was not detected in the MLCC used in our work, unlike other works [26]. This is likely because Pd was replaced by nickel in MLCC in the 1990s due to the rise in Pd price (Pd based MLCC are now used mostly in very specific high end medical applications), hence explaining the high weight ratio of Ni (66.1 w%) detected in the leaching solution. 

We then developed a hydrometallurgy process with the aim of minimizing the use of acid by improving the efficiency of leaching. To achieve this, we displaced the leaching reaction equilibrium by performing a simultaneous liquid–liquid extraction reaction, exploiting the non-miscibility and metallic affinity of IL Aliquat^®^ 336. Typically, the combined reaction took place within the same reactor (a centrifugation tube) containing both a hydrochloric acid (HCl) solution and an IL Aliquat^®^ 336 supernatant (1:1 in volume). Indeed, since metal ions are extracted into the organic phase simultaneously with the leaching process, it prevents the acidic aqueous solution from reaching a saturated state and avoids the occurrence of non-selective metal ion precipitation; furthermore, it displaces the equilibrium in favor of the metal leaching. Through a combined leaching and extraction process, the acid leaching’s performance can be maximized.

**Scheme 1:**
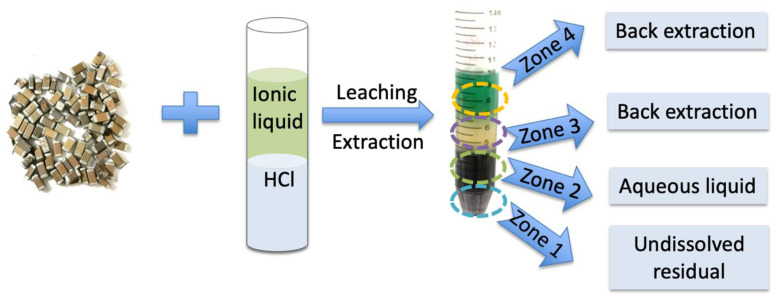
The experimental process of recycling metals from waste MLCC and the observed 4 domains (zone 1 = solid phase; zone 2 = aqueous phase; zones 3 and 4 = IL phases to be back-extracted).

Hence, after 5 h of lixiviation/extraction reaction under agitation, using an oscillator (1000 rpm), the bulk of the whole capacitor was dissolved and one could observe four different domains in the test tube (see Scheme 1): (i) a small amount of a powdered solid residue could be found at the bottom of the tube (zone 1); (ii) a liquid part made of three different zones, identified due to their different colors (Scheme 1), named zone 2 to 4. 

**Scheme 2:**
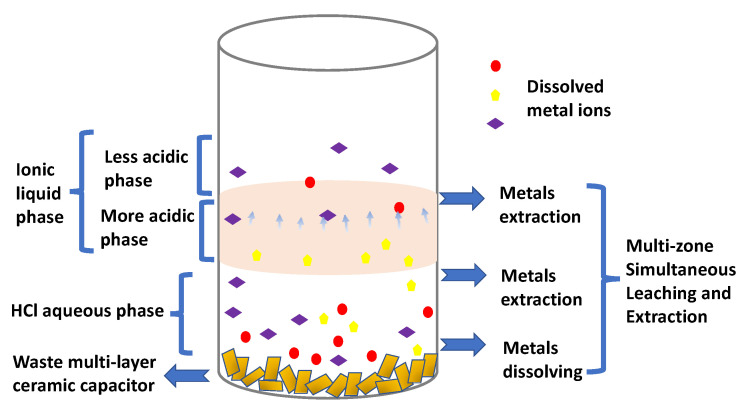
Simultaneous multi-zone leaching and extraction of valuable metals from waste MLCC by Aliquat^®^ 336-HCl-H_2_O system.

Firstly, the undissolved residue was dried completely, amounting to approximately 0.61 g (representing 59 wt% of the initial capacitor’s mass). Its composition was measured by SEM-EDX to be made of 43.6 wt% Ba and 10.9 wt% Ti elements. Other minority elements, such as chromium (3.8 wt%), copper (1.6 wt%), iron (1.1 wt%) and silicon (0.79 wt%), were also detected (Scheme 2 and Figure S2 in Supplementary Materials). Hence, this leaching protocol proves to be selective, as it allows for the recovery of a fraction enriched in Ba and Ti elements, when compared to the baseline composition.

In the liquid fraction (see Scheme 1), zone 2 comprised the HCl aqueous solution, which was delimited from zone 3 by a clear interface. The ICP-OES analysis performed indicated that Ni was its dominant metal element (85.0 wt%), which was more concentrated than the original value of 66.1 wt% reported above. It is also important to note that no copper was detected in the zone 2 solution, and only minority metals such as aluminum, nickel, cobalt, manganese, iron and lead made up the remaining 15 wt%. 

**Figure 1:**
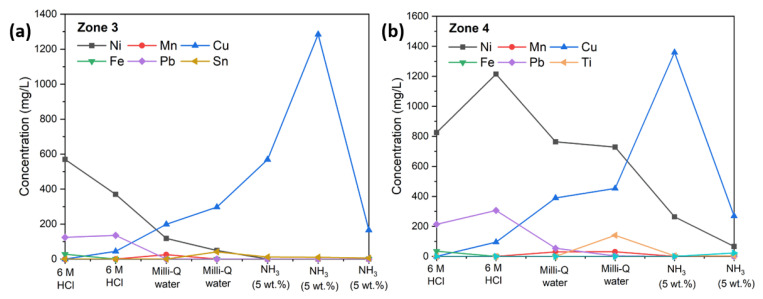
(**a**) Concentrations of metals after the multistep back-extraction process for zone 3. (**b**) Concentrations of metals after the multistep back-extraction process for zone 4.

Investigations on extracted metals in Aliquat^®^ 336 were conducted by multistep back-extraction using successively three different back-extraction agents [27]: (i) 6 M HCl; (ii) deionized water; (iii) 5 wt% aqueous solution of ammonia. Both zone 3 and zone 4 back-extraction results showed that Ni and Cu are the main elements extracted. The distribution coefficient of [Ni]_(Zone 4 + Zone 3)_/[Ni]_Zone 2_ between ionic liquids and the aqueous solution was 6.4; therefore, 86.5 wt% of Ni was extracted from the HCl aqueous phase using Aliquat^®^ 336 (Table 1). In zone 3, only a minute amount of Cu could be back-extracted using 6 M HCl, which increased significantly with increased pH until the maximum concentration of Cu was detected at 1280 ppm, when a 5 wt% aqueous solution of ammonia was used as the back-extraction agent. Zone 3 produced more pure Cu effluents, with the 5 wt% of ammonia agent contributing approximately 80 wt% of Cu with 98.6 wt% purity for the total amount of back-extracted Cu (Figure 1a). Using the same back-extraction agents and back-extraction procedures, zone 4 showed similar trends as zone 3 (Figure 1b), except more metals were stripped away in zone 4 than in zone 3. It is interesting to note that Ni was more favored in zone 4, where the distribution ratio, D_Ni_, between nickel’s concentration in zone 4 and zone 3 was 3.5. However, there was no difference for Cu between zone 4 and zone 3, as the D_Cu_ was 1 (see Table 1).

The mechanism of simultaneous multi-zone leaching and extraction process (Scheme 2) has been investigated as a function of time, with the position of the zone 3/zone 4 separation line moving upward with time (see Figure S3 in Supplementary Materials). After 5 h of lixiviation/extraction protocol, zone 3 and zone 4 were of similar sizes. We interpreted this overall phenomenon as being due to the migration of both the hydronium (H_3_O^+^) and metallic ions from the aqueous phase into the IL, leading to the separation of the two zones, delimitated by the migration front (no clear interface). It was also enhanced since the increased acidity of the IL changed its viscosity: the more acidic the IL, the lower its viscosity, which leads to a faster mass transfer [28]. Hence, the bottom section of the IL phase behaves similarly to the 6 M HCl solution, which has been proven to extract more metal ions. These findings are summarized in Scheme 2.

## 4. Metals Upcycling into Nanoparticles of Electrocatalyst

With sustainability and the overall recycling process cost in mind, it is key to limit as much as possible the number of purification steps. Hence, the ideal case is that an application can be found for some of the metal mixtures obtained, thereby avoiding further purification toward pure metals. Due to the easy separation and enrichment described above, we investigated using the Ni/Fe and Ni/Cu metal mixtures directly as precursors of electrocatalysts for the oxygen evolution reaction (OER) [29,30,31,32,33]. However, before these solutions could be used to synthesize electrocatalysts, Pb had first to be separated from the mixtures Ni/Fe and Ni/Cu found in the HCl back-extraction solutions of zones 3 and 4, which we successfully performed by selective precipitation using 6 M NaOH (see Table S2, Supplementary Materials for ion concentrations in HCl based back-extraction solutions obtained from zones 3 and 4 solutions before and after selective precipitation protocol). With this protocol, Pb stayed in the original solution and Ni/Fe and Ni/Cu mixtures could be recovered from dissolving precipitates obtained from zones 3 and 4, respectively. 

It should be noted that a large amount of catalyst must be synthesized to study its performance, and only a small amount of waste MLCC was recovered from discarded PCBs, so we performed the electrocatalyst synthesis using simulated solutions containing identical Ni/Fe or Ni/Cu concentrations to the HCl based back-extraction solutions obtained from zones 3 and 4, respectively. The hydroxide-based catalysts were synthesized by employing the propylene oxide mediated alkalization precipitation method that was previously reported for the synthesis of nickel layered double hydroxide (Ni-LDH, more details in Experimental Section), but modified to favor the making of amorphous nanoparticles to increase catalytic performances [34]. SEM, EDX and XRD analyses were performed on the obtained NiFe and NiCu-hydroxide electrocatalysts to study their morphologies, compositions and structures, respectively. 

**Figure 2:**
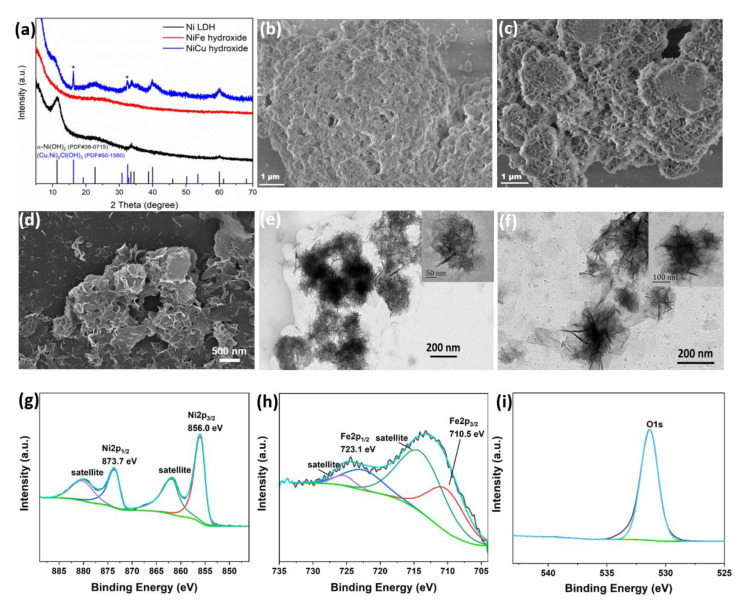
(**a**) The XRD patterns and SEM images. (**b**) NiFe-hydroxide, (**c**) NiCu-hydroxide and (**d**) Ni-LDH TEM images. (**e**) NiFe-hydroxide, (**f**) NiCu-hydroxide, (**g**) Ni 2p, (**h**) Fe 2p, and (**i**) O 1s XPS spectra.

The X-ray diffraction (XRD) patterns reported in Figure 2a showed some differences between the bimetallic hydroxide and Ni-LDH. The main peaks for the Ni LDH pattern are located at 11.4°, 22.7°, 33.7° and 59.9°. The pattern of the as-synthetized NiCu-hydroxide is consistent with the pattern of Ni-LDH, corresponding to α–Ni(OH)_2_ (PDF#38-0715), apart from two peaks marked with an asterisk (16.2° and 32.4°), which are attributed to the phase of copper nickel chloride hydroxide ((Cu, Ni)_2_Cl(OH)_3_, #PDF 50-1560). In contrast, the pattern of NiFe-hydroxide only showed a broad peak; no additional peak could be observed that would be indicative of a layered structure, as could be anticipated for an LDH. This indicates that the as-prepared NiFe-hydroxide is, at least from a structural point of view, mostly amorphous with a highly disordered structure, and therefore, structurally different from the Ni-LDH reference material, which was synthesized using the same method but without the addition of any Fe precursor. This amorphous structure is especially beneficial when NiFe-hydroxide is used as a nanoparticle-based electrocatalyst. Indeed, it allows for a high surface area and more exposed unsaturated atoms that are catalytically-active sites, which can enhance the OER performance [35,36]. Moreover, the SEM and TEM images revealed that the lateral size of NiFe-hydroxide nanosheets is much smaller than the Ni-LDH or even the NiCu-hydroxide (Figure 2b–f). This result is comparable to previous reports [23]. Finally, elemental mapping results obtained using SEM-EDX confirmed that the synthesized NiFe and NiCu hydroxides consisted of Ni/Fe/O and NiCu/O element mixtures, respectively (Figures S4 and S5, Supplementary Materials). The result of XPS spectra indicates the Ni^2+^ oxidation state of Ni sites in NiFe-hydroxide (Figure 2g). In Figure 2h, the two prominent doublet peaks are located at 710.5 and 723.1 eV corresponding to Fe 2p_3/2_ and Fe 2p_1/2_ for the Fe^3+^ oxidation state [36]. The main peak of O 1s XPS spectrum in Figure 2i is attributed to the O-H species in NiFe-hydroxide. The survey and C 1s XPS spectra of NiFe-hydroxide are also provided as Figure S6. For a better comparison, we also measured the XPS spectra of NiCu-hydroxide (Figure S7). The deconvolution of Cu 2p spectrum shows that the two doublet peaks are located at 935.1 and 954.6 eV corresponding to Cu 2p_3/2_ and Cu 2p_1/2_ for the Cu^2+^ oxidation state [37]. Noteworthy, the small doublet peaks at the low binding energy (932.2 eV Cu 2p_3/2_ and 952.1 eV Cu 2p_1/2_) may be ascribed to the Cu–Cl species from impurities [38].

**Figure 3:**
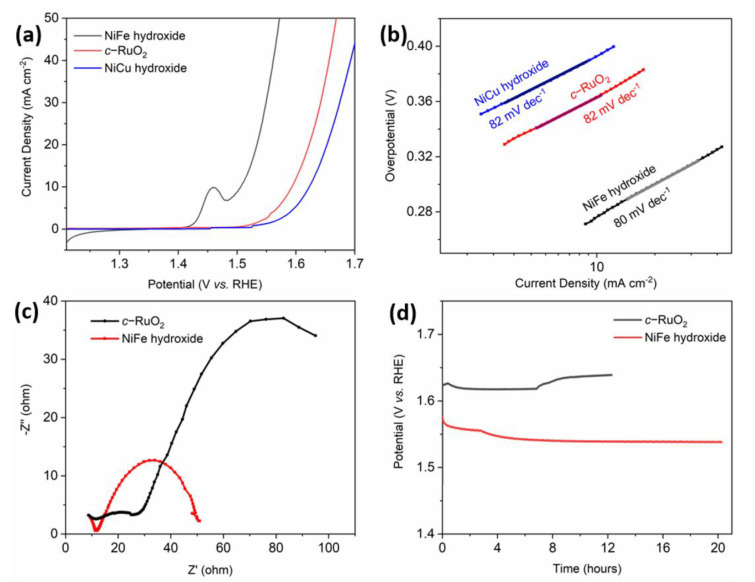
(**a**) Linear sweep voltammetry (LSV) curves of as-prepared nanoparticles of NiFe-hydroxide, NiCu-hydroxide and commercial c-RuO_2_ catalysts in 1 M KOH electrolyte. (**b**) The corresponding Tafel plots derived from LSV curves. (**c**) Nyquist plots recorded for NiFe-hydroxide and c-RuO_2_ at an applied potential of 1.50 V (vs. RHE). (**d**) Chronopotentiometric curve for NiFe-hydroxide and c-RuO_2_ at a constant current density of 20 mA cm^−2^ for more than 20 h in 1 M KOH.

To evaluate the OER performance of the NiFe-hydroxide electrocatalyst, a working electrode was prepared by depositing it on a glassy carbon electrode. Its electrochemical performance was investigated in 1 M KOH electrolyte using the three-electrode setup. As shown in Figure 3a, the polarization curves (no iR-correction) revealed that the NiFe-hydroxide electrocatalyst had excellent catalytic activity. This can be seen in the lower overpotential (303 mV) that is required to achieve a current density of 20 mA cm^−2^, which is better than those of the commercial RuO_2_ (c-RuO_2_) (390 mV) and NiCu-hydroxide (420 mV) electrocatalysts [39].

The Tafel plots that were derived from the polarization curves provided more in-depth insights on the OER kinetics. The linear portion of the Tafel plot was fitted using the Tafel Equation (5) [40]:

η = *b* log *j* + *a* (5)

where *j* refers to the current density, *b* refers to the Tafel slope and *a* refers to an intercept that is relative to the exchange current density. Based on the results shown in Figure 3b, we note that the NiFe-hydroxide electrocatalyst obtained a Tafel slope of 80 mV dec^−1^, and both the NiCu-hydroxide and commercial c-RuO_2_ obtained Tafel slopes of 82 mV dec^−1^. This suggests that the NiFe-hydroxide has slightly faster OER kinetics than the other two. To further validate a fast OER kinetic of electron transport for NiFe-hydroxide, the electrochemical impedance spectroscopy (EIS) was performed at an applied potential of 1.50 V (vs. RHE) (Figure 3c). The NiFe-hydroxide catalyst has a lower charge transfer resistance (R_ct_) than that of c-RuO_2_. Finally, the chronopotentiometry tests were also conducted in order to estimate the long-term durability of the NiFe-hydroxide catalyst. Figure 3c showed that its degradation is negligible when subjected to more than 20 h of catalysis in 1 M KOH at a constant current density of 20 mA.cm^−2^. In contrast, the c-RuO_2_ catalyst shows an obvious degradation after 7 h at the same current density.

## 5. Conclusions

In this work, simultaneous multi-zone leaching and extraction of valuable metals from waste MLCC using both hydrochloride acid (HCl) and IL Aliquat^®^ 336 has been successfully demonstrated. The multi-zone extraction process was also evaluated, and a mechanism was proposed. Concentrated and purified amounts mixtures of Ni and Cu were recovered from the back extraction process of each IL zone. In addition, recovered metal mixtures such as Ni/Fe and Ni/Cu were upcycled by the synthesis of amorphous electrocatalyst nanoparticles for OER, and their respective performances evaluated. In comparison to NiCu-hydroxide, NiFe-hydroxide is a better electrocatalyst, as it possesses faster OER kinetics. The latter even outperformed the reference and expensive electrocatalyst, c-RuO_2_, making the NiFe-hydroxide from recycled MLCC a good potential catalyst for OER.

## Figures and Tables

**Table 1:** Distribution ratios and separation factors of Ni and Cu in ionic liquid phase (zone 3 and zone 4) and aqueous phase (zone 2).

Name	[M]_zone4_/[M]_Zone 3_	[M]_zone4_/[M]_Zone 2_	[M]_zone3_/[M]_Zone 2_	[M]_Zone 4+ Zone 3_/[M]_Zone 2_
D_Ni_	3.5	5.0	1.4	6.4
D_Cu_^a^	1.0	-	-	-
*α*_Ni/Cu_	3.5	-	-	-

^a^ Cu in zone 2 was under the detection level of 1 mg/L.

## Data Availability

Available upon request.

## Supplementary Materials

The following supporting information can be downloaded at: https://www.mdpi.com/article/10.3390/nano12152697/s1, Figure S1. (a) The SEM image captured from the residual of leaching MLCC; (b) Energy disper-sive X-ray (EDX) elemental analysis of the residual of leaching MLCC, Figure S2. (a) SEM and (b) Energy dispersive X-ray (EDX) elemental analysis of the residual after MLCC leaching and extraction using the Aliquat^®^ 336-HCl-H2O system, Figure S3. Tube appearance evolution as a function of time during the MLCC combined leaching and extraction process (one MLCC placed in 1 mL of 6 M HCl and 1 mL Aliquat^®^ 336 agitated at 1000 rotations per minute (rpm) for 5 h at 60 °C, using the Eppendorf ThermoMixer C equipment), Figure S4. (a) SEM image of amorphous NiFe-hydroxide catalyst; (b), (c) and (d) the correspond-ing SEM-EDX element distribution images for O, Ni, and Fe. (e) The element composition of NiFe-hydroxide catalyst, Figure S5. (a) SEM image of NiCu-hydroxide catalyst. (b), (c) and (d) the corresponding SEM-EDX element distribution images for O, Cu and Ni. (e) The element composition of NiCu-hydroxide catalyst, Figure S6. XPS spectra for NiFe-hydroxide: (a) full spectra; (b) C 1s peak deconvolution, Figure S7. XPS spectra of NiCu-hydroxide: (a) full spectra, and various peaks deconvolution (b) C 1s, (c) O 1s, (d) Cu 2p and (e) Ni 2p, Table S1. Metals analysis of scrubbed and regenerated ionic liquid from Zone 3 and zone 4, Table S2. Metals analysis of Pb selective precipitation from the Ni, Fe and Pb and Ni, Cu, Pb HCl back-extraction solutions from Zone 3 (stream 1) and Zone 4 (stream 2).

## Abbreviations

MLCC, Multilayer Ceramic Capacitor; ICP-OES, inductively coupled plasma-optical emission spectrometry; SEM-EDX, field emission scanning electron microscope; IL, ionic liquid.
