# Supplementary material for: Efficient Electrocatalyst Nanoparticles from Upcycled Class II Capacitors
Source: Nanomaterials (Basel). 2022 Aug 5;12(15):2697. doi: 10.3390/nano12152697 (PMC9370706; doi:10.3390/nano12152697)
Supplement: Supplementary file 1 [file nanomaterials-12-02697-s001.zip › nanomaterials-1759034-supplementary.pdf]

nanomaterials

Supplementary Materials

Efficient Electrocatalyst Nanoparticles from Upcycled Class II Capacitors

Junhua Xu [1,5], Daobin Liu [1†], Carmen Lee [1], Pierre Feydi [2], Marlene Chapuis [2], Jing Yu [1,3], Emmanuel Billy [2], Qingyu Yan [1,3] and Jean-Christophe P. Gabriel [1,4,*]

[1] SCARCE Laboratory, Energy Research Institute @ NTU (ERI@N) | Nanyang Technology University , Singapore 637553, Singapore; junhua.xu@fjirsm.ac.cn (J.X.); ldbin@ustc.edu.cn (D.L.); carmen001@e.ntu.edu.sg (C.L.); yujing@ntu.edu.sg (J.Y.); alexyan@ntu.edu.sg (Q.Y.)
[2] LITEN | Université Grenoble Alpes, CEA, 38054 Grenoble, France; pierre.feydi@laposte.net (P.F.); marlene.chapuis@cea.fr (M.C.); emmanuel.billy@cea.fr (E.B.)
[3] School of Materials Science and Engineering, Nanyang Technological University, Singapore 639798, Singapore
[4] LICSEN | NIMBE| Université Paris-Saclay, CEA | CNRS, 91191 Gif-sur-Yvette, France
[5] Nuclear Chemistry & Separation and Purification Technology Laboratory, Fujian Institute of Research on the Structure of Matter, Chinese Academy of Sciences, Fuzhou, Fujian Province, 350002, China
[†] Current address: Institute for Solid State Physics, The University of Tokyo, Kashiwa, Chiba 277-8581, Japan
[*] Correspondence: jean-christophe.gabriel@cea.fr

(a)
(b)

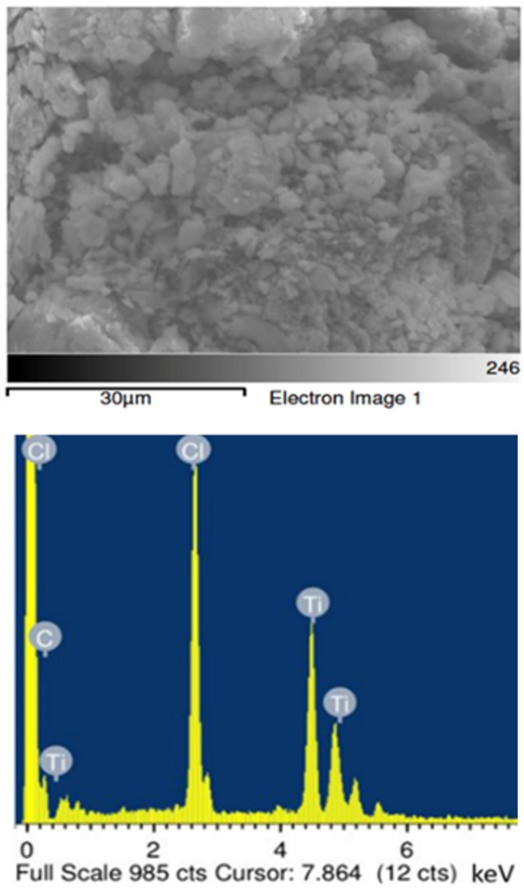

Figure S1. (**a**) The SEM image captured from the residual of leaching MLCC; (**b**) Energy dispersive X-ray (EDX) elemental analysis of the residual of leaching MLCC.

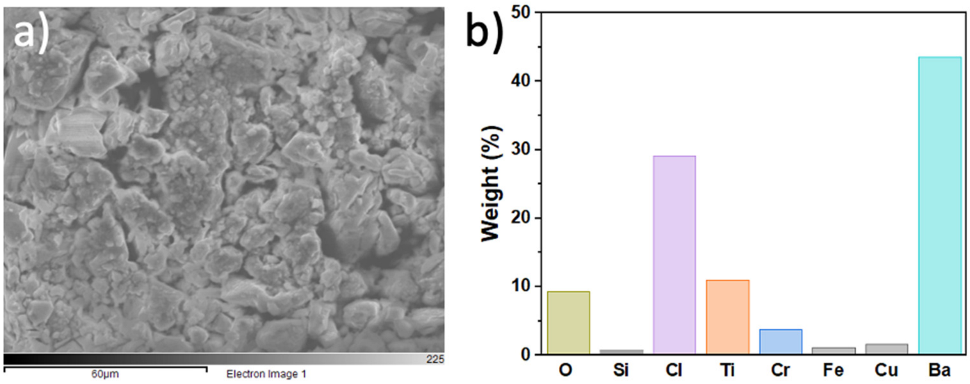

Figure S2. (**a**) SEM and (**b**) Energy dispersive X-ray (EDX) elemental analysis of the residual after MLCC leaching and extraction using the Aliquat® 336-HCl-H_2O system.

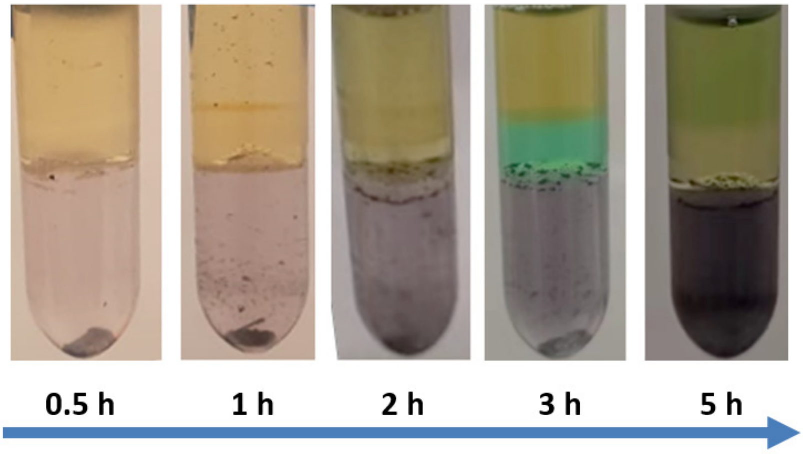

Figure S3. Tube appearance evolution as a function of time during the MLCC combined leaching and extraction process (one MLCC placed in 1 mL of 6 M HCl and 1 mL Aliquat® 336 agitated at 1000 rotations per minute (rpm) for 5h at 60°C, using the *Eppendorf ThermoMixer C* equipment).

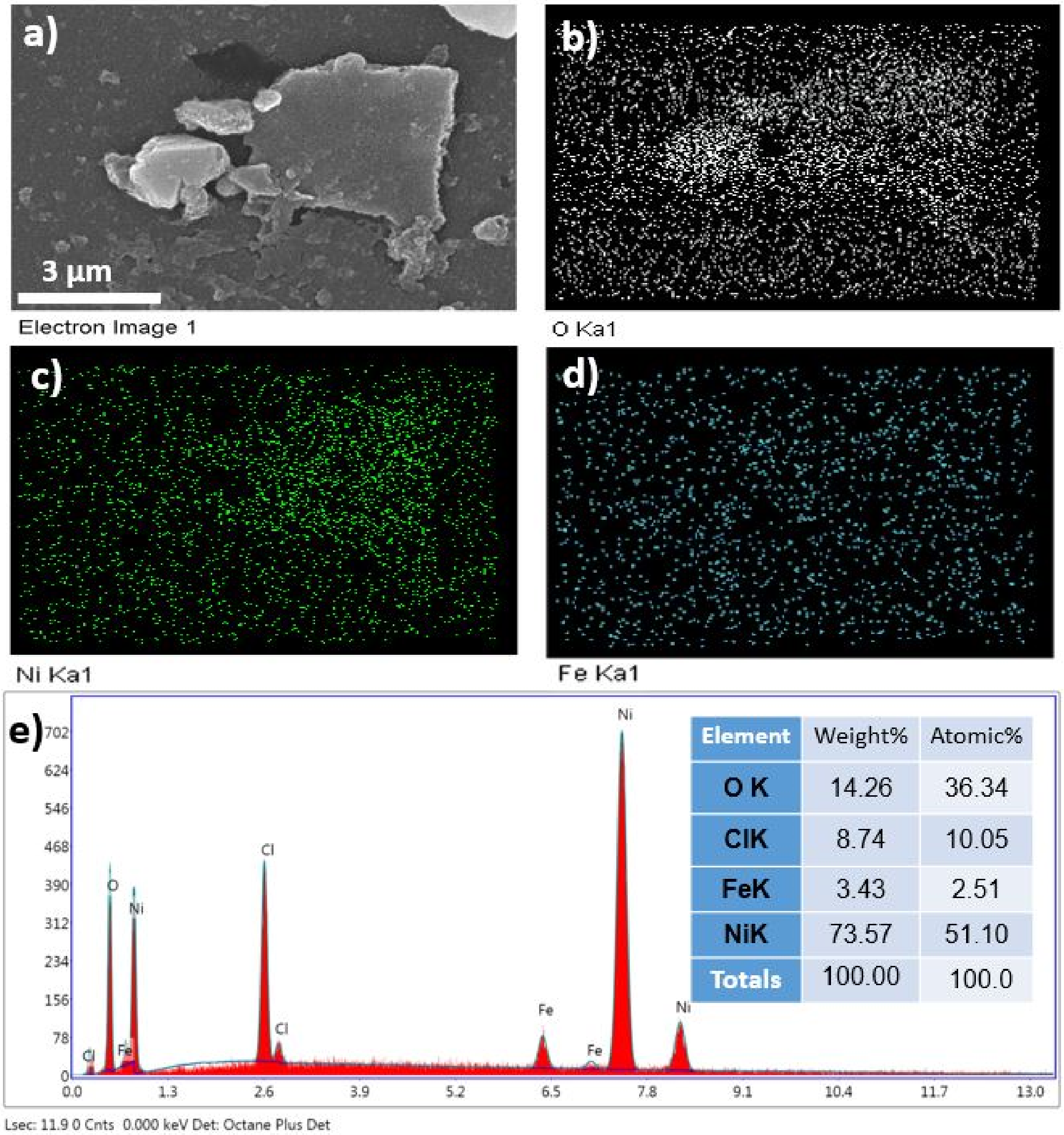

Figure S4. (**a**) SEM image of amorphous NiFe-hydroxide catalyst; (**b**), (**c**) and (**d**) the corresponding SEM-EDX element distribution images for O, Ni, and Fe. (**e**) The element composition of NiFe-hydroxide catalyst.

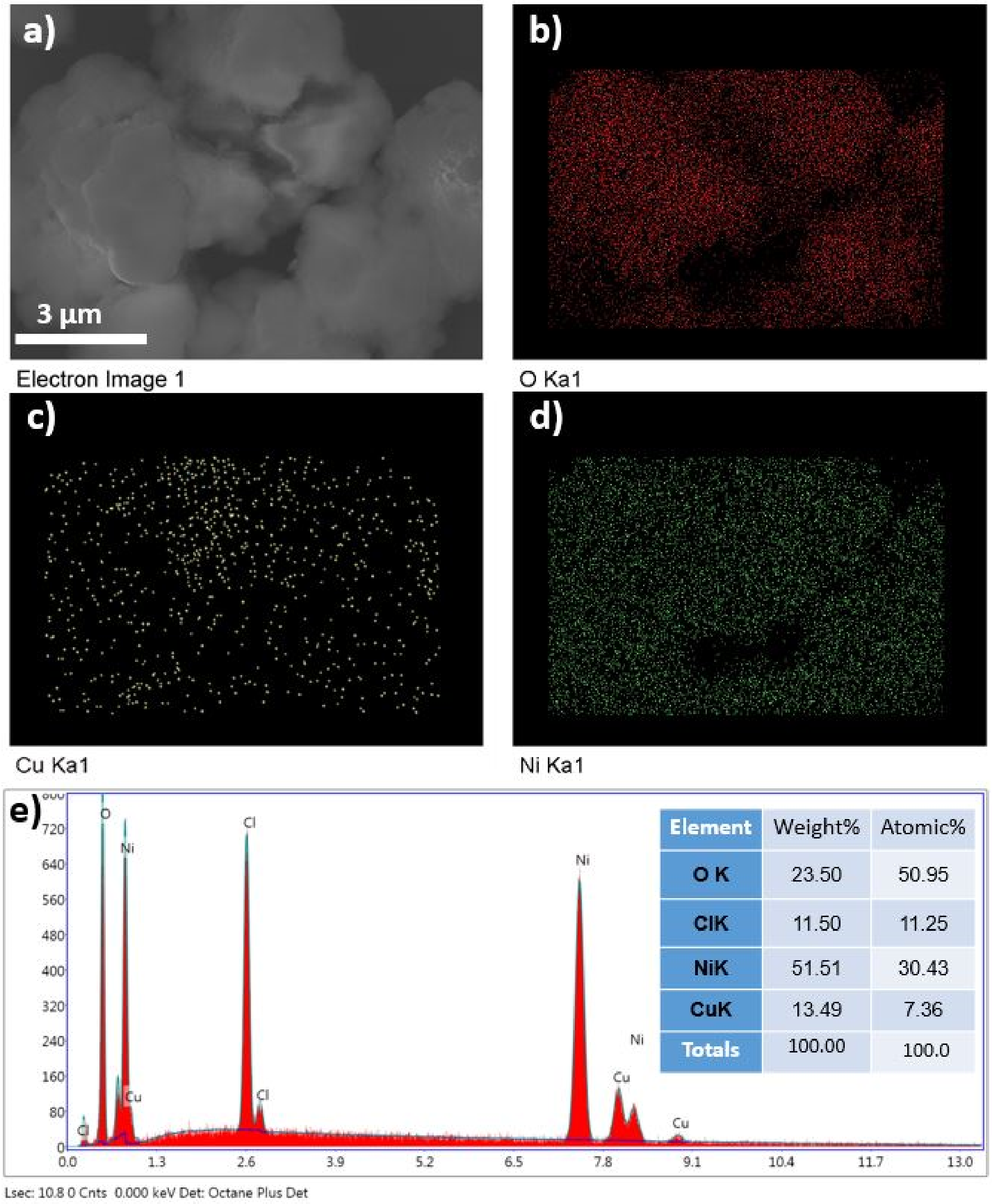

Element	Weight%	Atomic%
O K	23.50	50.95
ClK	11.50	11.25
NiK	51.51	30.43
CuK	13.49	7.36
Totals	100.00	100.0

Figure S5. (**a**) SEM image of NiCu-hydroxide catalyst. (**b**), (**c**) and (**d**) the corresponding SEM-EDX element distribution images for O, Cu and Ni. (**e**) The element composition of NiCu-hydroxide catalyst.

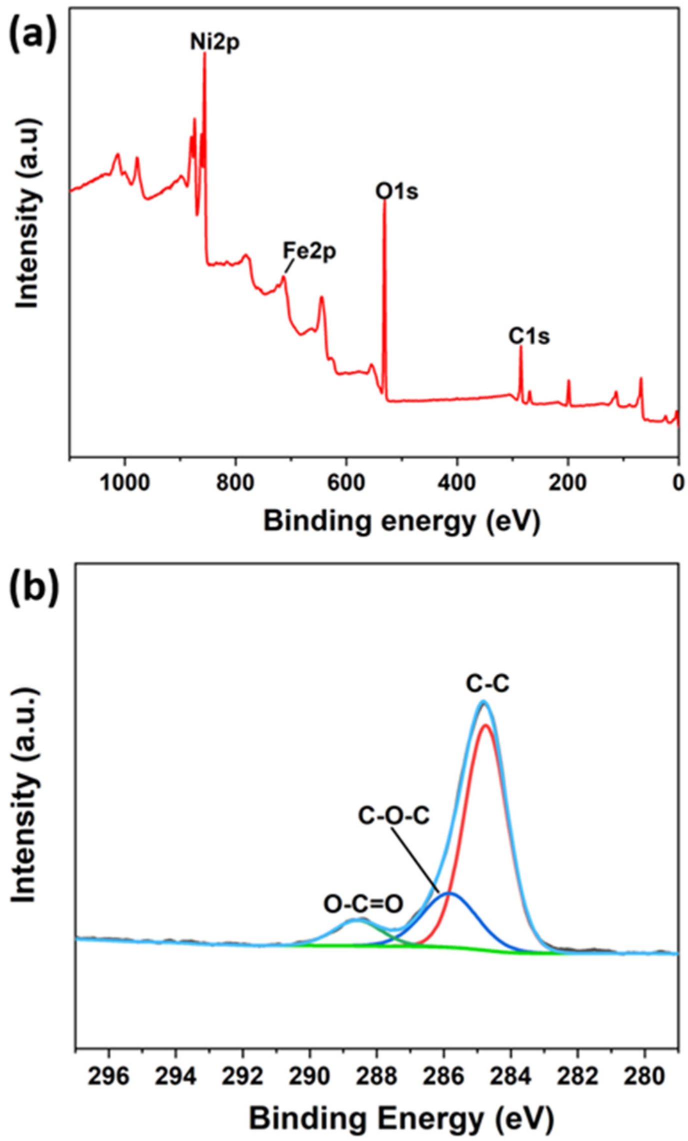

Figure S6. XPS spectra for NiFe-hydroxide: (a) full spectra; (b) C 1s peak deconvolution.

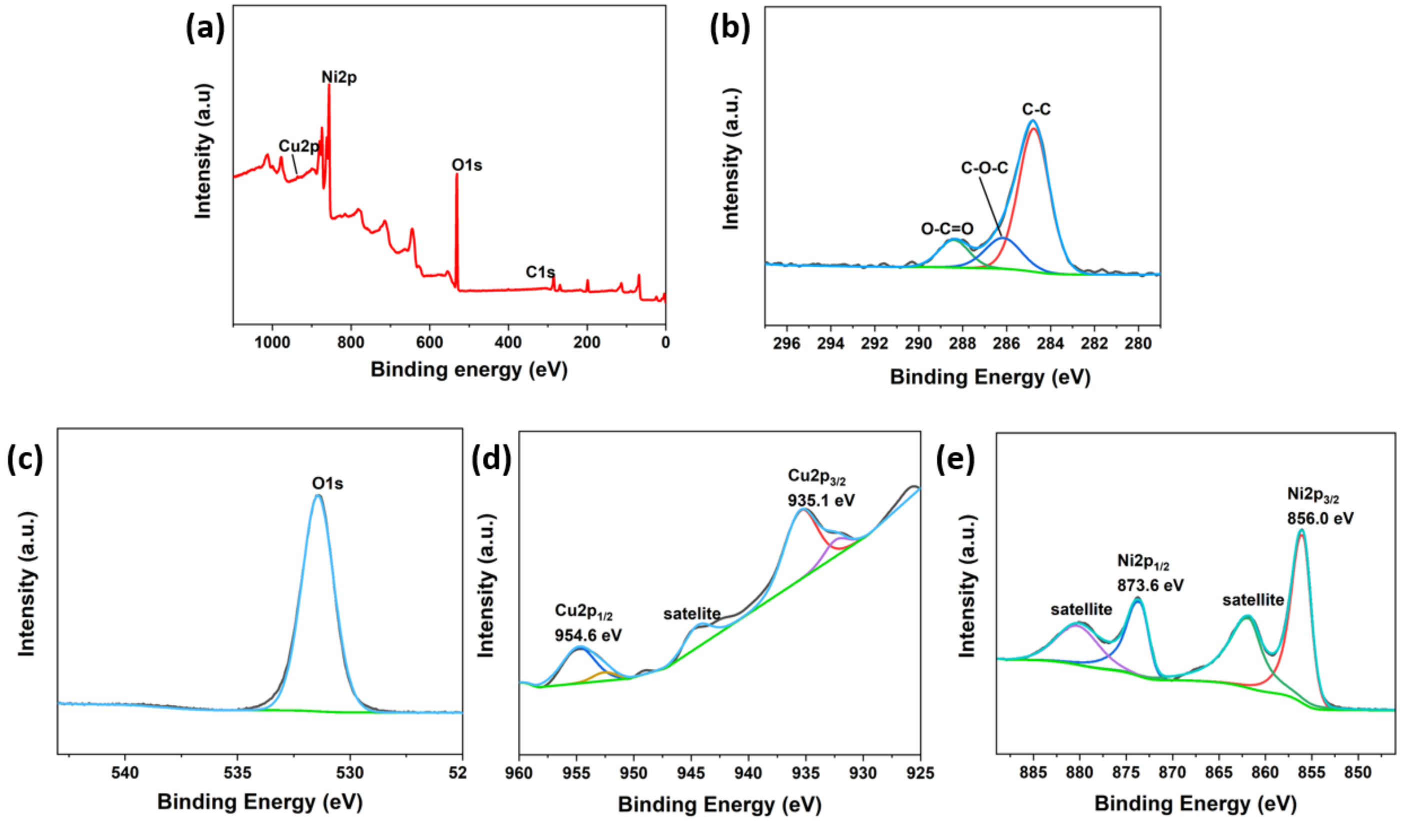

Figure S7. XPS spectra of NiCu-hydroxide: (**a**) full spectra, and various peaks deconvolution (**b**) C 1s, (**c**) O 1s, (**d**) Cu 2p and (**e**) Ni 2p.

Table S1. Metals analysis of scrubbed and regenerated ionic liquid from Zone 3 and zone 4.

	**Scrub Agent**			**Regenerate Agent**	
	6 M NaOH	6 M NaOH	6 M NaOH	0.5 M HCl	0.5 M HCl
Al	-	-	-	-	-
Ni	-	-	-	-	-
Zn	-	-	-	-	-
Co	-	-	-	-	-
Mn	-	-	-	-	-
Cu	57.3	6.4	-	-	-
Fe	-	-	-	-	-
Pb	-	-	-	-	-
Ti	-	-	-	-	-
Sn	350.1	272.2	76.8	-	-

Table S2. Metals analysis of Pb selective precipitation from the Ni, Fe and Pb and Ni, Cu, Pb HCl back-extraction solutions from Zone 3 (stream 1) and Zone 4 (stream 2).

**Solution**	**Metals**	**Original Solution (mg/L)**	**Solution After Precipitation (mg/L)**	**Solution Dissolved the Precipitation Solid by 1 M H_2SO_4 (mg/L)**
Stream 1	Fe	31.1	-	153.1
	Ni	697.3	-	3408.5
	Pb	169.1	37.37	-
Stream 2	Cu	69.7	-	350.5
	Ni	792.2	-	3892.9
	Pb	221.1	69.33	-
